# Rehabilitation Perspectives in Unilateral Upper Limb Peromelia: A Case Report

**DOI:** 10.7759/cureus.103194

**Published:** 2026-02-08

**Authors:** Kalyaanasundhary S, Gorle Sujatha, Smita Pathak

**Affiliations:** 1 Department of Physical Medicine and Rehabilitation, Jawaharlal Institute of Postgraduate Medical Education & Research, Puducherry, IND; 2 Department of Physical Medicine and Rehabilitation, Employees State Insurance Corporation-Post Graduate Institute of Medical Sciences and Research (ESIC PGIMSR) Basaidarapur, New Delhi, IND; 3 Department of Physical Medicine and Rehabilitation, All India Institute of Medical Sciences, Bhopal, Bhopal, IND

**Keywords:** congenital limb deficiency, peromelia, prosthesis, rehabilitation, transverse limb defect

## Abstract

We present the case of a two-year-old male child with unilateral right trans-radial peromelia, focusing on the rehabilitation strategy and the importance of early passive prosthetic fitting in enhancing functional and psychosocial outcomes. Peromelia, also referred to as congenital transverse limb deficiency, is characterized by truncation of a limb at varying levels due to arrested limb development. Management options for congenital limb deficiencies include prosthetic fitting and external stump lengthening, particularly in cases of trans-humeral and trans-radial peromelia. Early identification and timely prosthetic intervention are crucial, as they are associated with reduced prosthesis rejection rates and improved long-term functional outcomes. While cognitive maturity and the ability to participate in structured training are necessary for active prosthetic use, early passive prosthetic fitting plays a vital role in facilitating bimanual activities and preparing the child for future active prosthesis training.

## Introduction

Peromelia is a rare congenital anomaly characterized by transverse deficiency of the upper limb, most commonly at the trans-radial or trans-humeral level, resulting from disruption of the apical ectodermal ridge during limb development [1,2]. Although uncommon, this condition presents significant functional and psychosocial challenges for affected children and their families. Congenital upper-limb deficiencies are classified by the International Society for Prosthetics and Orthotics (ISPO) into transverse and longitudinal deficiencies based on the level and pattern of limb absence [3-5].

Early childhood is a critical period for the development of bimanual skills essential for activities of daily living. Children with unilateral upper-limb deficiency naturally rely more on their intact limb in the absence of early prosthetic or therapeutic intervention. This compensatory overuse can result in asymmetrical motor patterns, altered biomechanics, and potential overuse strain of the unaffected limb. Early prosthetic fitting, combined with bilateral training and rehabilitation, encourages more balanced use of both upper limbs, promotes symmetry, and reduces compensatory dominance of the intact limb [6-8]. Early prosthetic rehabilitation, particularly with passive prostheses, facilitates limb symmetry, promotes bimanual function, and supports psychosocial adaptation while preparing the child for future active prosthetic use. 

Prosthetic management in congenital upper-limb deficiency follows a developmental approach based on the child’s age, motor skills, and cognitive maturity. In infancy, passive prostheses are preferred mainly to promote body image, bilateral awareness, and participation in bimanual activities. During early childhood, as voluntary motor control improves, body-powered prostheses are introduced to enhance functional use. Myoelectric prostheses are generally considered in older children who demonstrate adequate cognitive ability, muscle control, and family support [6-8].

This case report highlights the rehabilitation approach and outcomes of early passive prosthetic fitting in a child with unilateral trans-radial peromelia.

## Case presentation

A two-year-old male child, born at full term by normal vaginal delivery with no significant antenatal history, was brought by his parents with a missing right hand below the elbow since birth. The child was examined and was diagnosed with right trans-radial peromelia with rudimentary digits. The parents were worried and anxious about the future of the child with regard to day-to-day activities.

**Figure 1:**
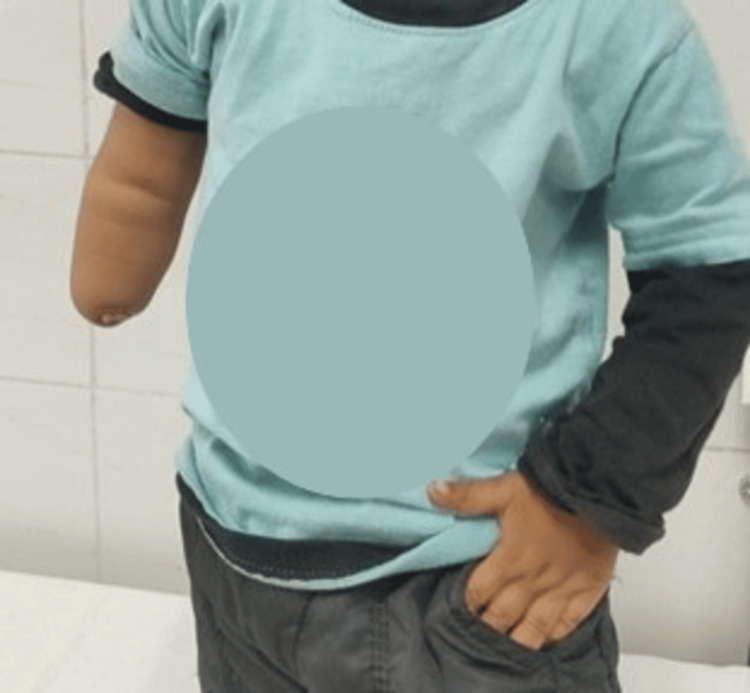
Child with right trans-radial elbow deficiency with rudimentary digits.

On examination, trans-radial deficiency with stump end dimpling and nubbin-like rudimentary digits was present, and the stump length was 7 cm from the elbow crease (Figure 1). The child demonstrated age-appropriate gross motor development with independent sitting, standing, and walking. Fine motor function was adequate for age, with effective use of the unaffected upper limb for grasping and manipulation, while the affected limb was used primarily for support and stabilization. There was active flexion and extension at the elbow and active shoulder movements, and the range of motion at the shoulder was full. According to Swanson's classification for congenital limb malformations, the child was classified to have type 1-failure of formation of parts at the trans-radial level [3].

For proper fitting of a prosthesis, the adequate length of the stump required is 6-7 cm from the elbow crease, along with sufficient power of the muscles and adequate range of movement of the joint [6]. As the stump was of adequate length, the child was prescribed a passive below-elbow prosthesis with Munster socket, forearm shell with cosmetic hand for adaptation, and to train for use of an active prosthesis at a later age. The prognosis was explained to the child’s parents along with the treatment plan. 

The prosthesis was custom-fabricated by a certified prosthetist and orthotist using a self-suspending Munster socket, which is a self-suspending trans-radial prosthetic socket that provides suspension by contouring around the medial and lateral epicondyles of the humerus. This design improves stability, reduces skin irritation, and eliminates the need for external straps or harness systems. The socket does not need any extra suspension attachment. The inner socket was made of thermoplastic material, with an external polyurethane foam forearm shell and a cosmetic hand. The inner liner of the Münster socket was fabricated using a thermoformable plastic sheet to ensure a customized, comfortable, and secure fit. The outer forearm shell consisted of polyurethane foam covered with cotton gloves to provide a natural appearance for the cosmetic hand. 

**Figure 2:**
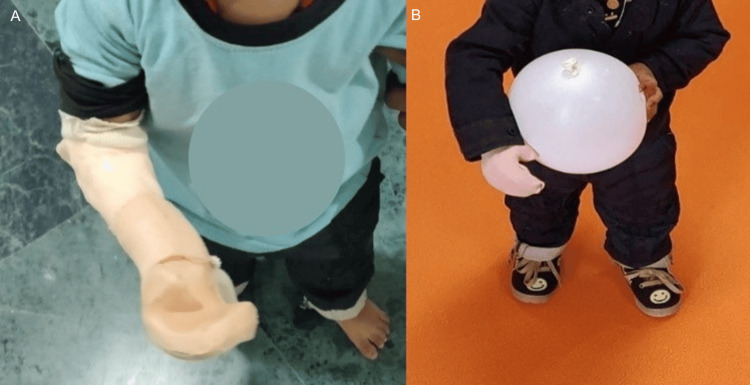
(A) The patient is fitted with a passive prosthesis; (C) using the passive prosthesis to hold and stabilise objects

Prosthetic fit and measurements were reviewed every three to six months to accommodate growth and ensure comfort, as recommended in pediatric prosthetic practice. The child was advised graded use during waking hours, especially during play and functional activities. He demonstrated good acceptance and used the prosthesis for support and object stabilization, with no complications noted during follow-up. He was able to use the prosthesis for bimanual activities such as stabilizing objects, holding toys, assisting during play, and supporting daily activities appropriate for his age (Figure 2).

## Discussion

Parents and caregivers should be counseled regarding gradual prosthesis use, regular wearing schedules, and the importance of positive reinforcement during daily activities. Early incorporation of the prosthesis into play and routine tasks improves acceptance and functional use. Regular follow-up is essential to monitor fit, skin condition, and growth-related changes. Psychological support and reassurance to family members help address anxiety and promote long-term adherence to prosthetic use.

Any congenital abnormality is distressing news for the parents and family members along with the child, which may lead to profound grief in them. They are also worried about the impact of the deformity on the physical and psychosocial ability of the child [7]. The main goal of treatment in such cases is to restore the appearance and function of the limb to as normal as possible and to provide a sense of support to parents by explaining to them the benefits of prosthesis usage to the child.

In the present case, there was an absence of the right upper limb, which is in accordance with the study by Wynne-Davis et al. [10], which states that transverse deficiency is almost always unilateral.

A child who is missing one hand will find difficulties in sit-to-stand activities and other activities of daily living. Ideally. Developmental studies consistently show that between five and six months of age, infants begin purposeful grasping and transfer of objects between hands, reflecting the emergence of early bimanual coordination and the functional importance of bilateral upper-limb use [11-13].

Providing the child with a prosthesis helps in maintaining symmetric limb length and enables the child to hold toys and objects with both hands, and facilitates easy manipulation. A study by Davids et al. revealed improved prosthetic outcomes in a child who was fitted with a prosthesis before three years of age than at a later age [8]. Also, fitting with a temporary prosthesis makes the child and parents understand that it is a permanent part of the body used in replacement of the actual part, and helps in fitting an active prosthesis at a later stage.

A passive prosthesis provides a natural appearance and enhances it. Formal quantitative outcome measures were not used in this case due to the child’s very young age and the limited feasibility of structured testing. Assessment was primarily clinical and functional, based on observation of prosthesis acceptance, comfort, bimanual use, and caregiver feedback during pre- and post-fitting follow-up visits, self-image, along with psychological and functional advantages. The role of observational functional assessment in toddlers has been supported by existing pediatric rehabilitation literature [11]. This enables the child to forget the disability and have a good social life. The primary goal of treatment is to help the child have a normal growth and development pattern and enable them to be independent [14]. Studies have shown that early prosthetic fitting in a unilateral trans-radial deficiency is a strong indicator of a child’s continued wear of the prosthesis later in life, whereas fitting at an older age results in prosthesis rejection [15].

## Conclusions

Early prosthetic fitting in children with unilateral upper-limb deficiency has been associated with improved psychosocial well-being, self-esteem, and social integration, even when immediate functional benefits are limited. The present case is noteworthy as it supports the role of early passive prosthetic fitting in facilitating bimanual activities, enhancing psychosocial health, and preparing the child for subsequent functional prosthetic use. The child was fitted with a temporary passive prosthesis to enable him to do bimanual activities, for social acceptance, to maintain limb symmetry, and to avoid overuse injuries. Following the fitting of the prosthesis, the child was smiling and was able to do bimanual handling, thus proving that it will have a positive impact on the psychosocial health of the child. This report emphasizes a rehabilitation-focused approach to early prosthetic intervention in unilateral upper-limb peromelia. It highlights the role of early passive prosthetic use in promoting bimanual function, psychosocial adaptation, parental acceptance, and readiness for subsequent active prosthetic training. It also provides practical clinical insights for rehabilitation physicians involved in the management of similar cases during early childhood. Therefore, early intervention should be considered whenever feasible to optimize developmental progress and psychosocial outcomes.
